# Free anterolateral thigh flaps for upper extremity soft tissue reconstruction

**DOI:** 10.3205/iprs000064

**Published:** 2015-02-05

**Authors:** Nick Spindler, Sammy Al-Benna, Andrej Ring, Heinz Homann, Lars Steinsträsser, Hans-Ulrich Steinau, Stefan Langer

**Affiliations:** 1 Abteilung für Plastische, Ästhetische und Spezielle Handchirurgie, Klinik für Orthopädie, Unfallchirurgie und Plastische Chirurgie, Universitätsklinik Leipzig, Deutschland; 2 Department of Plastic, Reconstructive and Burns Surgery, Nottingham City Hospital, Nottingham, United Kingdom; 3 Klink für Plastische Chirurgie und Schwerbrandverletzte, Berufsgenossenschaftliches Klinikum Bergmannsheil Bochum, Deutschland; 4 Klinik für Handchirurgie, Plastische Chirurgie und Brandverletzungen, Berufsgenossenschaftliche Unfallklinik Duisburg, Deutschland; 5 Universitätsklinik für Plastische-, Rekonstruktive und Ästhetische Chirurgie, Handchirurgie, Evangelisches Krankenhaus Oldenburg, Deutschland; 6 Klinik für Unfallchirurgie/Plastische Chirurgie, Sarkomzentrum am Westdeutschen Tumorzentrum, Universitätsklinikum Essen, Deutschland

**Keywords:** free tissue flaps, reconstructive surgical procedures, upper extremity, wounds and injuries

## Abstract

**Introduction:** Limb-threatening wounds of the upper extremity pose a challenge to the micro vascular surgeon. The aim of this study is to analyze the outcome of free anterolateral thigh flaps for upper extremity soft tissue reconstruction.

**Methods: **A retrospective review of patients undergoing this procedure from 2005 to 2012 was performed. Case note analysis was performed to determine demographic and perioperative factors, and complications and outcomes.

**Results: **Thirty-two patients with a mean age of 53 years (9–84 yrs) underwent upper extremity reconstruction with an anterolateral thigh (ALT) flap. There were 24 (75%) males and 8 (25%) females. The etiology of the soft tissue defects was: infection (44.6%); post-tumor ablation (40%); and trauma (15.6%). The defect site was most commonly in the forearm (53.1%), followed by the elbow (12.5 %), arm (12.5%) and hand (21.9%). The mean timing of free flap transfer was 6.8 days after admission to our institution (minimum 1 days, maximum 9 days). Mean operative time of surgery was 4 h 39 min (minimum 3 h 2 min, maximum 6 h 20 min). The mean hospitalization was 24.8 days (minimum 5, maximum 85). The ALT success rate was at 92.3%. Partial flap necrosis was documented in five cases (15.6%). Complete flap loss occurred in two post-traumatic cases who both lost their limbs.

**Discussion:** This flap, in the hands of experienced surgeons, provides reliable coverage of upper extremity defects.

## Zusammenfassung

**Einleitung: **Nach Trauma oder tumorresezierenden Eingriffen stellen die Extremitäten gefährdende Wunden der oberen Extremität immer wieder eine mikrochirurgische Herausforderung in der rekonstruktiven Chirurgie dar. Neben schnellem und suffizientem Verschluss der Wunde sollte die Rekonstruktion unter optimalen ästhetischen und insbesondere unter funktionellen Gesichtspunkten geplant und durchgeführt werden.

**Methoden: **Es erfolgte eine retrospektive Analyse, der im Zeitraum von 2005 bis 2012 am Bergmannsheil Bochum plastisch chirurgisch versorgten Patienten, welche mittels eines freien anterolateralen Oberschenkel-(ALT-)Lappens an den oberen Extremitäten gedeckt wurden.

**Ergebnisse: **Zwei Drittel der mittels ALT an der oberen Extremität versorgten Patienten waren durchschnittlich 53 Jahre alt (9–84 Jahre). 24 (75%) Patienten waren männlich, 8 (25%) weiblich. Defektursache waren in 44,6% der Fälle Infektionen der Weichteile, in 40% Weichteildefekte nach Tumorresektionen und in 15,6% posttraumatisch bedingt. In 53,1% der Fälle waren die Defekte am Unterarm lokalisiert, in 12,5% am Ellenbogen und in 21.9% an der Hand. 6,8 Tage nach Zuverlegung in unser Krankenhaus erfolgte die plastische Versorgung mittels freiem ALT-Lappen (min. 1 Tag, max. 9 Tage). Die durchschnittliche OP-Zeit lag bei 4 Stunden und 39 Minuten. (min. 3 h 2 min, max. 6 h 20 min). Die mittlere Verweildauer betrug 24,8 Tage (min. 5 Tage, max. 85 Tage) Die Erfolgsrate nach freier ALT-Lappendeckung betrug 92,3%, in 5 Fällen (15,6%) kam es zu einer partiellen Lappennekrose. Ein kompletter Lappenverlust zeigte sich bei 2 posttraumatisch versorgten Patienten, bei diesen musste im Verlauf die Extremität amputiert werden.

**Diskussion: **Der freie ALT-Lappen ist in der Hand des erfahrenen plastischen Chirurgen eine sichere Deckungsoption an der oberen Extremität. Die Hebedefektmorbidität ist gering, der Lappen bietet einen langen Gefäßstil mit einer verlässlichen Hautinsel und ist somit eine sichere Deckungsoption für Weichteilwunden an der oberen Extremität.

## Introduction

Radical debridement allows the surgeon to prevent infection by skilful use of the scalpel and is the necessary foundation for the microsurgical techniques of applying free flap coverage [1]. Free tissue transfer is warranted when local flaps cannot be harvested outside the zone of injury or when soft tissue defects are extensive and involve exposed vital structures such as bone, tendons, nerves, and vessels. Other major indications for free flap coverage include coverage following tumor ablation or soft tissue infections. Most importantly, these benefits include the ability to provide composite tissue coverage and reconstruction of all damaged or missing tissues and early recovery of function. Microsurgical reconstruction has improved substantially over the last three decades. Microsurgery has evolved to focus on functional and aesthetic results, as well as donor site morbidity. The anterolateral thigh (ALT) flap is a fasciocutaneous (FC) flap based on the septocutaneous or musculocutaneous perforators of the descending branch of the lateral circumflex femoral artery, first described by Song in 1984 [2]. It is well established in Asian countries, where it has also first emerged and has been in regular use ever since [3]. As a result of its great success, the ALT has become an increasingly popular technique in Europe [4], [5], [6]. To achieve better cosmetic results, bulky flaps can be thinned out during primary or in following second procedure [7], [8], [9]. Over the years the ALT flap has become the workhorse for reconstruction of skin and soft tissue defects and especially over the past decade, replacing many other flaps [6], [7], [8], [9], [10].

## Materials and methods

A retrospective descriptive study of all patients who underwent upper extremity reconstruction with an ALT flap admitted to the BG University Hospital Bergmannsheil, over a period of 7 years (2005–2012). Demographical data, data regarding the surgical procedure itself, the postoperative period as well as follow-up data will be presented and discussed.

### Anatomy

A satisfactory perforator is generally found within a range of 3 cm of the midpoint of a line connecting the superolateral border of the patella with the anterior superior iliac spine. More than half of the perforators traverse the substance of the vastus lateralis muscle. The descending branch of the lateral circumflex femoral artery, and its committing veins, lies between the vastus lateralis and rectus femoris muscles, along with the nerve to the vastus lateralis. The descending branch can usually be safely dissected proximally to its major branch to the rectus femoris, which should be preserved during flap harvest.

In theory, almost the entire anterolateral side of the thigh can be harvested as a skin paddle. The skin paddle is designed slightly larger than the exact defect size in order to minimise tension yet to permit primary closure of the donor site. In most cases, the fascia lata represents the deep aspect of the harvested flap, but suprafascial harvest is possible. Portions of vastus lateralis muscle can be harvested with the flap if necessary. The position of the nerve to vastus lateralis is variable with respect to the vascular pedicle.

### Surgical technique

Find the midpoint of a line drawn between the anterior superior iliac crest and the superolateral border of the patella. Use a handheld Doppler probe pre- and intra-operatively (as there is a change in perforator position from erect or supine) to identify a cutaneous perforator, located within 3 cm of the midpoint [6], [10], [11]. Additional perforators may be present distally or proximally. Design an elliptical flap centred at the main perforator. Skin paddle width should be based on the defect. The length should be sufficient to obtain closure without standing cutaneous “scooped-out” deformities – excess skin can be easily trimmed during inset. The medial side of the skin paddle is incised, with a proximal extension toward the anterior superior iliac spine. The incision is taken down to the fascia lata, and then the fascia is incised. The recognition of the rectus femoris is critical, and is sometimes difficult in debilitated patients. It is identified by its bipennate morphology (“chevron pointing up”). The fascia is raised off the rectus femoris, taking care to identify the cutaneous perforators which are visualized through the fascia. The rectus femoris is then retracted medially to expose the descending branch of the lateral circumflex femoral artery in the base of the intermuscular septum. The course of the cutaneous perforator is then assessed, and additional perforators can be identified. The nerve to the vastus lateralis is identified. If the perforators are musculocutaneous, they should be uncovered at this stage, to their junction with the descending branch of the lateral circumflex femoral vessels. This is accomplished by meticulous dissection with fine scissors, bipolar cautery and small vessel ligation clips. Circumferential flap dissection should not be performed at this stage. When all perforators have been uncovered, the lateral skin incision is made and carried through the fascia lata. Working from inferior to superior, the fascia lata (and skin paddle) is dissected off the underlying vastus lateralis until the most inferior perforator is reached. This perforator is then released from its surrounding muscle, again using meticulous dissection and small vascular clips. If a single perforator is to be used, a small amount of muscle should be left adherent to it, in order to allow identification of twisting of the pedicle. This is not necessary if two or more perforators are included in the flap. The remaining perforators are released in the same manner as the first. Perfusion through the perforators should be verified by checking for bleeding at the cut skin edges of the flap. It is imperative that the flap/pedicle is not to be placed under torsion or the pedicle may become occluded during harvest. The descending branch of the lateral circumflex femoral artery and vein are then dissected superiorly to their branches to the rectus femoris, which should be preserved. The pedicle is then divided.

For an upper extremity reconstruction, especially in the obese patient, the flap should be trimmed to be thin. It can be as thin as 3 mm without compromising the viability of the skin flap. A circle of fascia around the perforator is incorporated. The flap can be large for coverage of the forearm, especially for extensive volar defects with tendon exposure. It may preserve subcutaneous tissue, which facilitates future procedure of tendon transfers. Wrist, hand and thenar web: The FC-ALT flap has been used for coverage of traumatic cases. The trauma cases can be divided into acute and chronic groups. For acute, traumatic defects the flap should be larger than the measured defect to prevent later swelling of the wound; primary thinning of the flap is not advised because of the situation of contamination. For a chronic case of trauma, an ALT flap can be trimmed to as thin as 3 mm. It is often used for coverage after release of contracture. For the thenar web, however, proper volume can be preserved at its central portion to offer a better aesthetic result. Closure was performed by undermining medially and laterally in the suprafascial plane. The fascia lata was not re-approximated. Suction drainage was placed. The skin is closed in two layers.

## Results

32 patients with a mean age of 53 years (9–84 yrs) underwent upper extremity reconstruction with an ALT flap at our institution hospital. There were 24 (75%) males and 8 (25%) females (Table 1 (Tab. 1)). The aetiology of the soft tissue defects was: infection (44.6%); post-tumour ablation (40%); and trauma (15.6%). The defect site was most commonly in the forearm (53.1%), followed by the elbow (12.5 %), arm (12.5%) and hand (21.9%). Flaps were harvested from the right thigh in 44% of the cases and from the left thigh in 56 % of the cases. All donor sites were closed primarily. The mean timing of free flap transfer was 6.8 days after admission to our institution (minimum 1 days, maximum 9 days). Mean operative time of surgery was 4 hours 39 min (minimum 3 h 2 min, maximum 6 h 20 min). The mean hospitalization was 24.8 days (minimum 5, maximum 85). The ALT success rate was at 92.3%. There were postoperative complications in 31.3% of the cases. In four cases (12.5%) a revision of the arterial or venous anastomosis was necessary. Partial flap necrosis was documented in five cases (15.6%). Complete flap loss occurred in two post-traumatic cases who both lost their limbs. The limb salvage rate was 93.8% in this series. No donor site morbidity was noted. In the postoperative period, no infection, no hematoma, nor patient’s mortality were noted (Table 2 (Tab. 2), Table 3 (Tab. 3)).

## Case examples

### Case 1 – Reconstruction post-trauma

**Figure 1:**
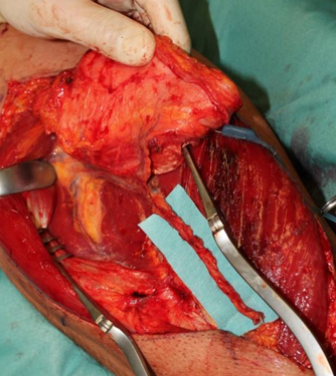
Harvested ALT flap

**Figure 2:**
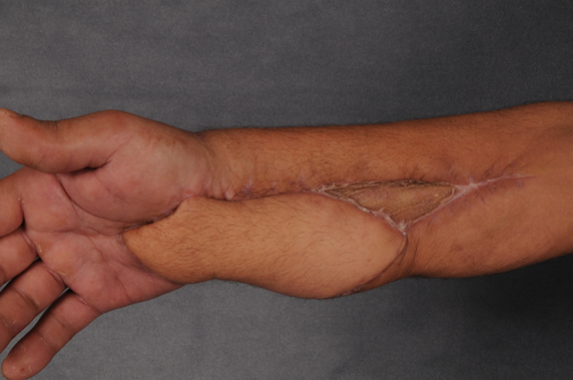
Postoperative outcome 12 weeks after surgery

A 54-year-old male sustained a crush injury to his right forearm and a cold hand. Exposure of multiple flexor tendons and large devitalized tissue on the ulnar flexor area were noted. During the initial examination, no pulsation was noted at the distal part of the limb. Capillary refill >2 sec was checked. The hand appeared pale and cold. Digital subtraction angiography was arranged immediately. It revealed transection of ulnar artery. His ulnar nerve and median nerve had also been injured. Initial resuscitation was performed at admission. An aggressive debridement was carried out. All devitalized flexor tendons, nerves, ligaments, muscles and vessels were removed radically. A flow-through free FC-ALT flap, 15 cm × 8 cm in size including partial tensor fascia lata with a 14 cm long pedicle was harvested from the left leg for wound coverage which was nourished by the descending branch of LCFA (Figure 1 (Fig. 1)). The flap was composed of 3 perforators. All of the vessels’ quality for anastomosis were checked under microscopic magnificent. The proximal end of the ulnar artery was anastomosed to the descending branch of LCFA. The distal end of the descending branch of the LCFA was anastomosed to the distal end of the ulnar artery. In addition, one concomitant vein of the ulnar artery was anastomosed to a concomitant vein of the descending branch of LCFA and the proximal end of this concomitant vein was anastomosed to the cephalic vein for the flap’s venous drainage. The distal hand immediately turned warm and pink after revascularization through the flow-through arterial anastomosis. Heparinisation formula was administrated for five days. The flap’s circulation was satisfactory postoperatively. CTA study was arranged 3 weeks after surgery. The stable flow-through arterial system was established from proximal ulnar artery to distal ulnar artery. The limb was salvaged. Neither donor nor recipient site morbidity was noted during the follow-up period. The patient was discharged after 20 days (Figure 2 (Fig. 2)).

### Case 2 – Reconstruction post soft tissue infection

**Figure 3:**
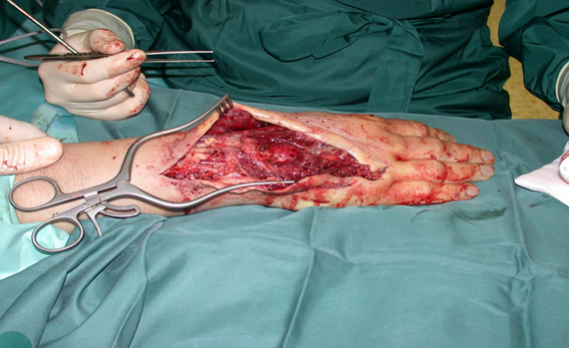
Infectious case with large dorsal defect of the hand. After wound debridement an ALT flap will be chosen to cover the defect

**Figure 4:**
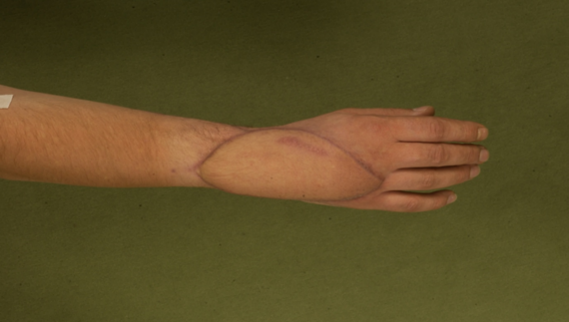
Identical patient showing postoperative outcome of the ALT. The previous large dorsal hand defect has been sufficiently covered by the ALT. The flap may be thinned out in a following secondary procedure

A 57-year-old female suffered soft tissue loss of the forearm and hand after a cortisone injection into the carpometacarpal joint of her right hand (Figure 3 (Fig. 3)). She underwent four separate debridements prior to transfer to our hospital. After one further debridement at our hospital, the wound was clean and reconstructed two days later with a FC-ALT flap raised from the left thigh. The flap artery was anastomosed to the radial artery in an end-to-end-technique. The intra- and postoperative development showed no complications. Neither donor nor recipient site morbidity was noted during the follow-up period. After a week of immobilisation, the patient was allowed to start slow and careful physical therapy as well as manual lymph drainage. The patient was discharged after 8 days. Already in the early follow up controls the patient presented a good function as well as a cosmetically satisfying outcome (Figure 4 (Fig. 4)).

### Case 3 – Reconstruction post-tumour ablation 

A 62-year-old female was diagnosed with a recurrent G2 myofibroblastic sarcoma (UICC grade III) on her right proximal extensor forearm invading her radius bone. The tumour was ablated with curative intent. The bone defect was reconstructed with vascularized bone grafts from the iliac crest. The soft tissue defect was covered using a free FC-ALT flap harvested from the left thigh. The proximal end of the ulnar artery was anastomosed to the descending branch of LCFA. In addition, one concomitant veins of the ulnar artery was anastomosed to a concomitant vein of the descending branch of LCFA for the flap’s venous drainage. Neither donor nor recipient site morbidity was noted during the follow-up period. The patient was discharged after 4 days. There was no tumour recurrence over the next three years and the patient was satisfied with both functional and aesthetic results.

## Discussion

All surgeons have to face the challenge of the complex management of devastating injuries to upper extremities. To do so, they should immediately evaluate the patient’s systematic condition, the patency of vascularity, and the area of damaged tissue at the acute stage. Limb salvage, functional recovery, and aesthetic appearance can be optimised if primary reconstruction can be performed appropriately. Early wound debridement and free flap reconstruction can protect underlying healthy vital structures, allow limbs salvage, and improve functional and aesthetic results [2], [3], [6].

Patients in this series had the presence of both large soft tissue defects and some (e.g., case 1) had varied segmental main artery defects with compromised circulation at distal upper limb. The advantage of the flow-through free ALT flap transfer is that it provides vascular conduit and soft tissue coverage simultaneously. In addition, artery-to-artery anastomoses are more conformable than vein grafts as the bridge, and reducing the number of anastomosis lowers the possible risk. The ALT flap is reliable and suitable for flow-through fashion [12], [13]. The long pedicle length meets the requirement if there is a need to establish the vascular continuity by flow-through fashion in the scenario of long vascular segmental loss. Meanwhile, characteristics of large soft tissue coverage ensure the anastomosis site can be covered safely and outside the zone of trauma. The anatomic variation and steep learning curve is considered a disadvantage [12], [13], [14]. In our series, flow through ALT flaps provided optimal solution for simultaneous large soft tissue coverage and re-establishment of vascular integrity. The selection of the recipient vessels, either radial or ulnar artery, depends on the position of the flap and its vessel’s anatomy. The recipient vessels should be sizable, in good quality, and far from the zone of trauma.

In upper extremity reconstruction, the idea still prevails that muscle flaps are a prerequisite to cope with infection. There is little or no substantial evidence though to prove the superiority of muscle flaps compared with well-vascularized fasciocutaneous flaps. Preservation of the large branch vessels ensures flap survival during primary defatting, allowing for improved contour in single-stage reconstructions [10].

The success rate of 92.3% in this series is similar to other studies [1]. In the cases who had flap failure in our series, even though intraoperative microscopic inspection of the patency of vessels was performed precisely, the extent and accurate zone of trauma was difficult to be evaluated initially and amputations were required. Mean operative time of surgery was 4 h 39 min in this series. Prolonged anaesthesia time (>10 h) may lead to the development of significant systematic complications. The two team approach and no need for position change can effectively decrease operation time [6], [15].

For the patients with compromised circulation, early establishment of adequate blood supply for limb salvage is also a priority. However, early flap coverage is not always possible in certain situations. Patients with co-morbid problems, such as cardiovascular, neurosurgical, renal functional, and haemodynamic problems, must be optimised prior to surgery which may delay the intention of the early free tissue coverage of the upper extremities [1].

### ALT donor site advantages

After three decades of development, the advantages of the fasciocutaneous ALT (FC-ALT) flap are clear [6] and can be classified as follows: Flap dissection from the donor site: (A) Relatively constant anatomy for easy and safe dissection of the flap. Difficult cases of anatomical variations occur in only 2% to 3% of patients. Strategies have been developed to overcome these problems. In an experienced hand, the success rate can reach almost 100%. (B) Large and long pedicle, easy and secure for anastomoses. (C) Minimal donor site morbidity. The motor weakness is mild and transient, and the donor site can be closed primarily if the width of the flap is less than 8 cm [14].

### ALT recipient site advantages

For the recipient site: (A) versatility in chimeric composite design because of sufficient tissue volume. It can be large (as large as half of the surface of the thigh) or small, thick or thin (as thin as 3 mm to 5 mm), with various tissue components based on one pedicle (large and pliable cutaneous area, the fascia lata can be used to repair tendons, the vastus lateralis muscle can functionally restore muscle defects, and incorporation of osseous components created by anastomoses to the distal end of the descending branch of lateral femoral circumflex artery is also possible); (B) Good texture, except for the hair growth in some male patients; (C) Can be reinnervated to become a sensate flap when including the lateral femoral cutaneous nerve; (D) Easy debulking procedure as there is no large artery in the subcutaneous layer as in the forearm flap and the option to trim the flap to be thin and become suitable for application to the forearm and thenar web; (E) Suppleness with structure provided by a solid aponeurosis; (F) The two team approach and no need for position change can effectively decrease operation time; (G) The advantage of creating a flow-through-flap to ensure the circulation of the extremity.

The main drawback of the ALT flap in German patients is thickness. The adipose panniculus of the thigh is thick even in thin subjects, and especially in women. Thinning at harvesting has been suggested, but is not always advisable, as this threatens skin paddle vascularisation and increases the rate of complications; it is more often performed secondarily. It is important to note that the thickness of the flap has a substantial influence on the necessary flap width to close the defect [16]. Although we did not always perform massive thinning in our patients, the thickness of the flap can be adjusted to individual needs during primary surgery by removing the deep fascia and subcutaneous fat, except for a 2 to 3 cm area around the entry of the vascular pedicle into the flap. Thus, the flap can be harvested as a thinned skin flap even in extremely fat patients. Therefore, by adapting the thickness and extension of the soft tissue defect, the pliable property of the skin can permit reconstruction with a functionally and aesthetically acceptable structure. In this series, direct closure of the donor defect was possible, even up to 12 cm in width.

Although the perforator vessel can be musculocutaneous or septocutaneous, it is isolated in mostly musculocutaneous form in about three-quarters of cases, depending on the series. Doppler is a reliable and inexpensive examination. It is widely used, but does not precisely determine perforator trajectory or, in particular, definitively distinguish septal and muscular variants or assess muscular trajectory length [11], [12].

Immediate reconstruction of severe upper extremity injuries favours increased function, fewer complications, shorter hospital stays, and thus lower costs compared with delayed reconstruction. Restoring blood supply to the area allows the immune system to expedite the healing of damaged tissues [17]. Immediate reconstruction also permits earlier mobilization, which helps to maintain the ROM of the joints and prevents the tendon adhesions that usually accompany delayed repair [1]. In this series, rehabilitation began as soon as 24 hours after surgery, before muscles lost significant strength or excursion.

## Conclusion

In conclusion, although there are some variations in its vascular pedicle, which derives irregularly from the main vessels and involves minimal morbidity of the donor site, the ALT flap offers the following advantages for an upper extremity defect: harvesting the flap is easy and rapid, especially when surgeons have gained experience with its use; it has a long and large vascular pedicle; it has a reliable cutaneous skin paddle, and its volume can be adjusted by trimming the flap to be thin and become suitable for application to the forearm and thenar are keys to the successful transfer of the flap for immediate reconstruction of upper extremity defects [6]. As in most other soft tissue defects that have been reported in the literature recently, with its evident functional and aesthetic advantages, the ALT flap can be considered an excellent and ideal alternative to the most commonly used conventional methods for most soft tissue defects [6].

## Notes

### Competing interests

The authors declare that they have no competing interests.

## Figures and Tables

**Table 1:**
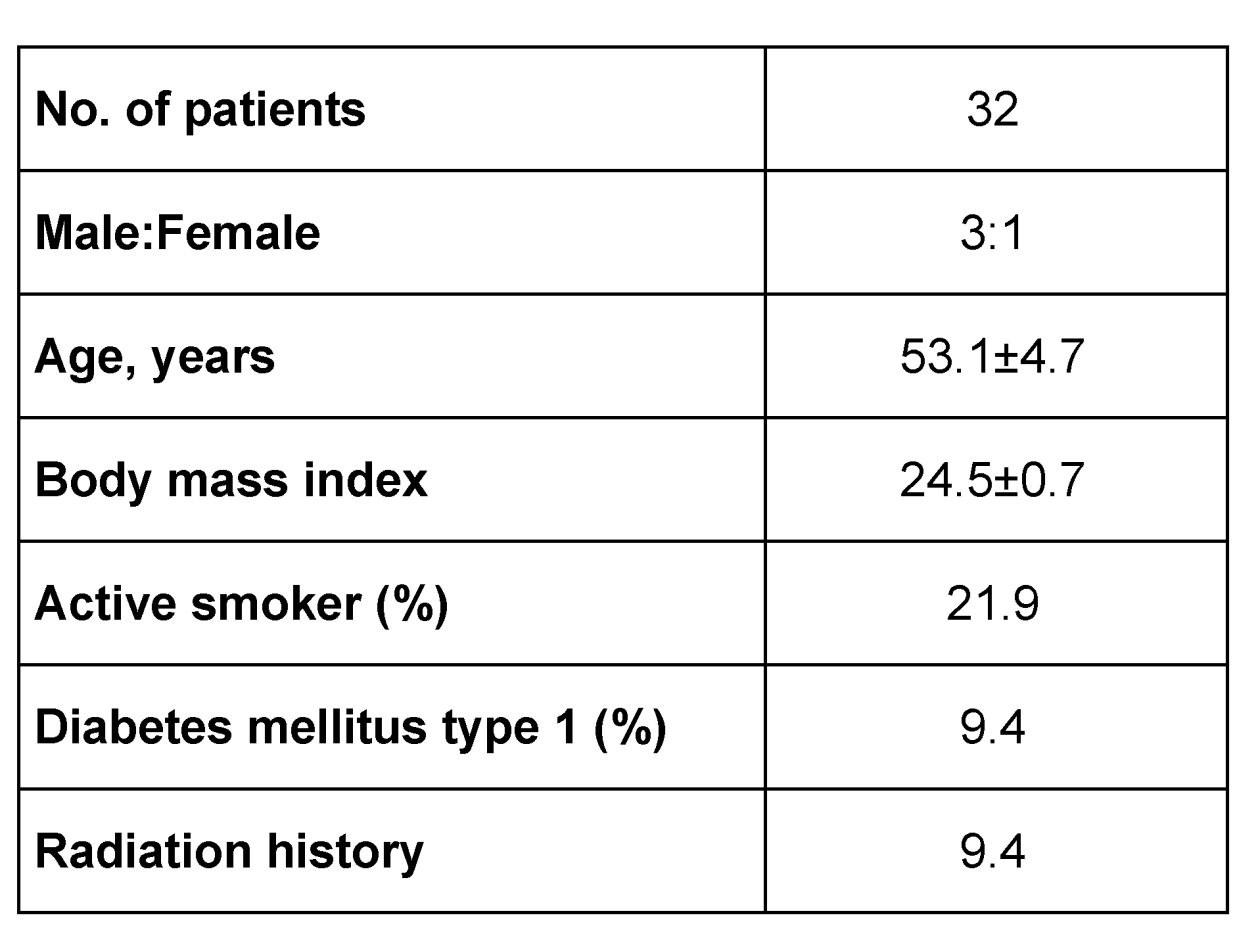
Patient demographics and history. Results are presented as mean±SEM.

**Table 2:**
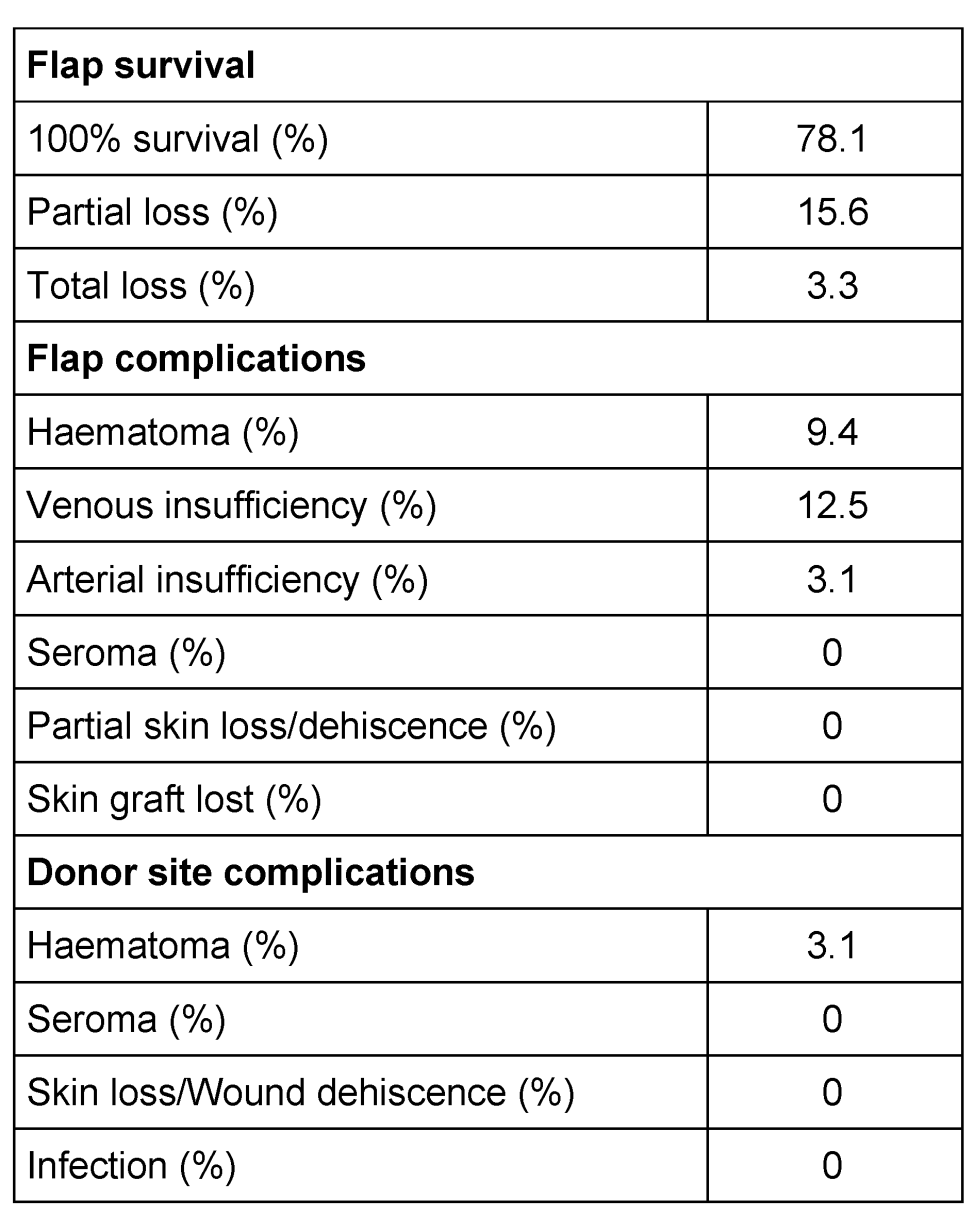
Complications following FC-ALT flap reconstruction

**Table 3:**
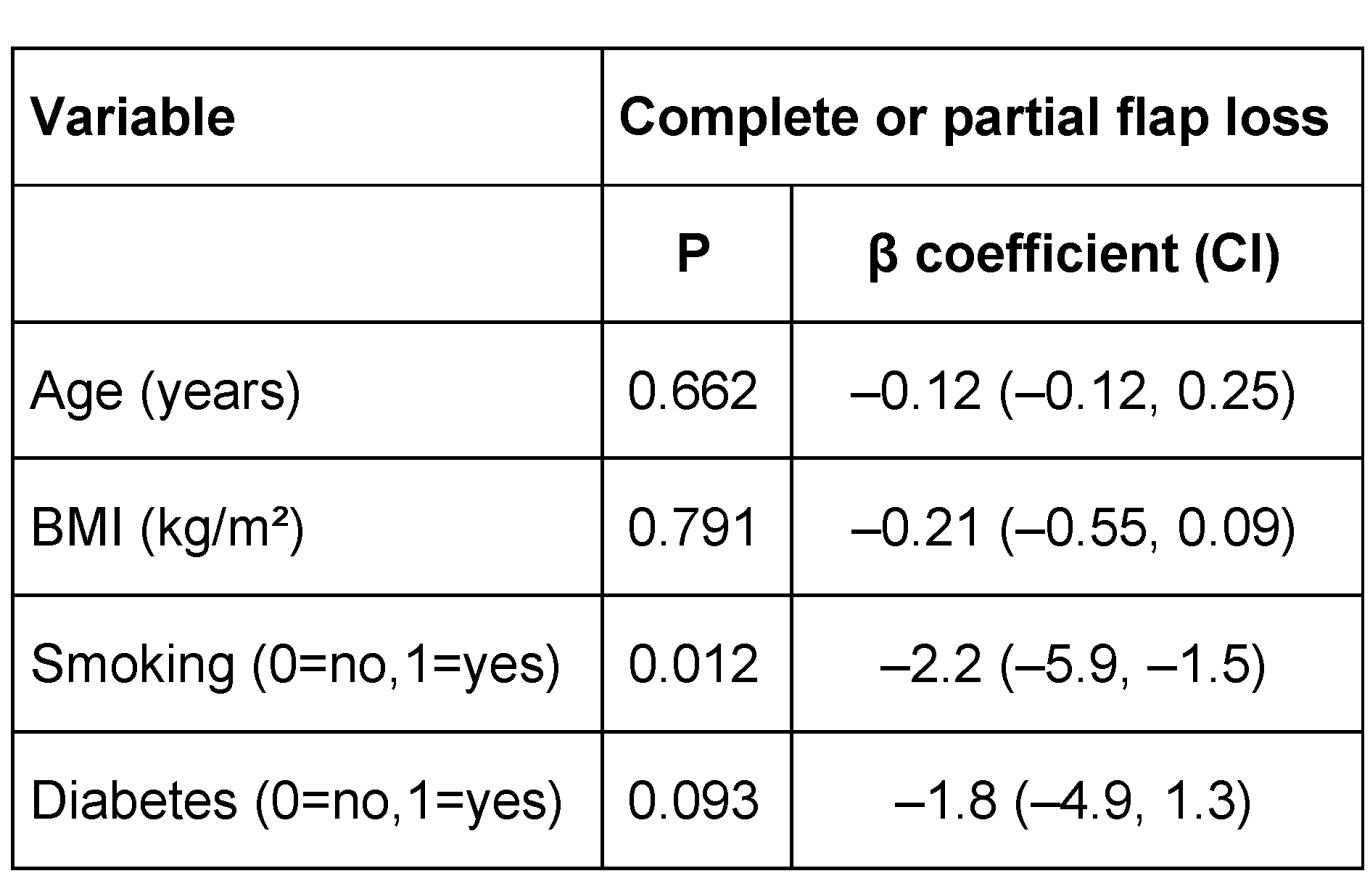
Analysis of the relationship between risk factors and flap loss (complete or partial) in patients undergoing upper extremity reconstruction with FC-ALT flaps. Analysis of variance p-values for the null hypothesis of no relationship between risk factors and complete or partial flap loss are displayed. Regression coefficients (β coefficient) and their 95% confidence intervals (CI) for the risk factors are also given. The coefficient of determination (R^2^) for this analysis is 13.3%. BMI, Body mass index. Smoking refers to current active smoking. Diabetes refers to type 1 diabetes mellitus.
